# Pre-clinical investigation of liquid sirolimus for local drug delivery

**DOI:** 10.3389/fcvm.2023.1184816

**Published:** 2023-09-15

**Authors:** Meagan Todd, Linda B. Liu, Justin M. Saul, Saami K. Yazdani

**Affiliations:** ^1^ Department of Engineering, Wake Forest University, Winston-Salem, NC, United States; ^2^ Department of Chemical, Paper and Biomedical Engineering, Miami University, Oxford, OH, United States

**Keywords:** sirolimus, liquid, *ex vivo*, pharmacokinetics, drug delivery, peripheral arterial disease, delivery device

## Abstract

**Introduction:**

Sirolimus is currently being explored as an alternative drug to paclitaxel for the treatment of peripheral artery disease (PAD). To date, sirolimus has only been used as drug coatings for stents and balloons and no studies have yet demonstrated the delivery of sirolimus in liquid form. The purpose of this pilot study was to investigate the feasibility of the delivery of liquid sirolimus into arterial segments in a benchtop peripheral artery bioreactor.

**Methods:**

The feasibility to deliver liquid therapy was first tested on four drug delivery devices using a fluorescently tagged liquid drug and an *ex vivo* porcine artery benchtop model. The four devices included the Bullfrog micro-infusion device, ClearWay RX catheter, Occlusion perfusion catheter (OPC), and the targeted adjustable pharmaceutical administration system (TAPAS). Penetration of the fluorescently tagged drug was measured via microscopic imaging and quantification of the depth of drug penetration into all device-treated tissue. Based on the penetration outcome, we then selected a single device to deliver liquid sirolimus into the *ex vivo* porcine artery model undergoing physiological flow and pressure conditions. The liquid sirolimus-treated arteries were collected from the *ex vivo* bioreactor at 1- and 24-hour post-delivery and arterial drug retention analyzed by liquid chromatography-tandem mass spectrometry.

**Results:**

Fluorescent microscopy demonstrated that drug delivery with the OPC had greater drug penetration into the medial wall as compared to other devices (OPC: 234 ± 161 µm; TAPAS: 127 ± 68 µm; ClearWay: 118 ± 77 µm; Bullfrog: 2.12 ± 3.78 µm; *p* = 0.098). The results of the *ex vivo* flow-circuit bench top model showed that the OPC device successfully delivered the liquid sirolimus at 1-hour (5.17 ± 4.48 ng/mg) and 24-hour (0.78 ± 0.55 ng/mg).

**Conclusions:**

These results demonstrate for the first time the ability to deliver liquid sirolimus directly to the medial layer of an artery via a liquid delivery catheter.

## Introduction

Percutaneous interventional therapies, including drug-eluting stents (DESs) and drug-coated balloons (DCBs), are the preferred approach in the treatment of peripheral artery disease (PAD) (1). To date, paclitaxel has been the most common anti-proliferative drug utilized in DES and DCBs, but recently, the use of the anti-proliferative drug sirolimus (also known as rapamycin) to treat PAD lesions is being explored as an alternative to paclitaxel (2). Unlike paclitaxel, sirolimus reversibly binds to FKBP12, a subunit of the TGF-β1 receptor, forming a sirolimus-FKBP12 complex that inhibits the activity of kinase mammalian target of rapamycin (mTOR). This inhibition of mTOR prevents cell cycle progression from the G1 phase to the S phase, making it a cytostatic drug, as opposed to paclitaxel which is a cytotoxic drug that irreversibly binds to microtubules (3, 4).

The use of sirolimus for non-stent platforms such as DCBs has gained momentum in recent years primarily because of concerns raised against the safety of paclitaxel. Up to now, all FDA-approved DCBs are coated with the crystalline form of paclitaxel. This crystalline formulation increases the retention and pharmacokinetics of the drug in arterial tissue but reduces its solubility (5). Additionally, the solid particles pose the risk of embolizing to distal organs and tissue, thereby affecting more than the target lesion. Sirolimus provides a potentially safer alternative due to its reversible binding on mTOR (6).

In addition to selection of safer anti-proliferative drugs, newer delivery approaches have explored alternative approaches in local delivery of therapeutics to the target lesion. These include a variety of liquid drug delivery devices designed for the treatment of PAD. Recently, both clinical and preclinical studies have shown that the liquid form of paclitaxel can be successfully delivered to the medial layer of arterial tissue using liquid delivery devices (5, 7). However, to date, little investigation into the delivery of liquid sirolimus has been explored.

In this study, we investigated the feasibility of delivering the liquid form of sirolimus into arterial tissue. We first evaluated the delivery efficacy of four liquid drug delivery devices—the Bullfrog micro-infusion device, ClearWay RX catheter, Occlusion perfusion catheter (OPC), and the targeted adjustable pharmaceutical administration system (TAPAS) into arterial tissue. The delivery and retention of dissolved sirolimus using a liquid delivery device was then evaluated in a clinically relevant *ex vivo* model mimicking physiological flow conditions.

## Materials and methods

### *Ex vivo* bioreactor system

**Figure 1:**
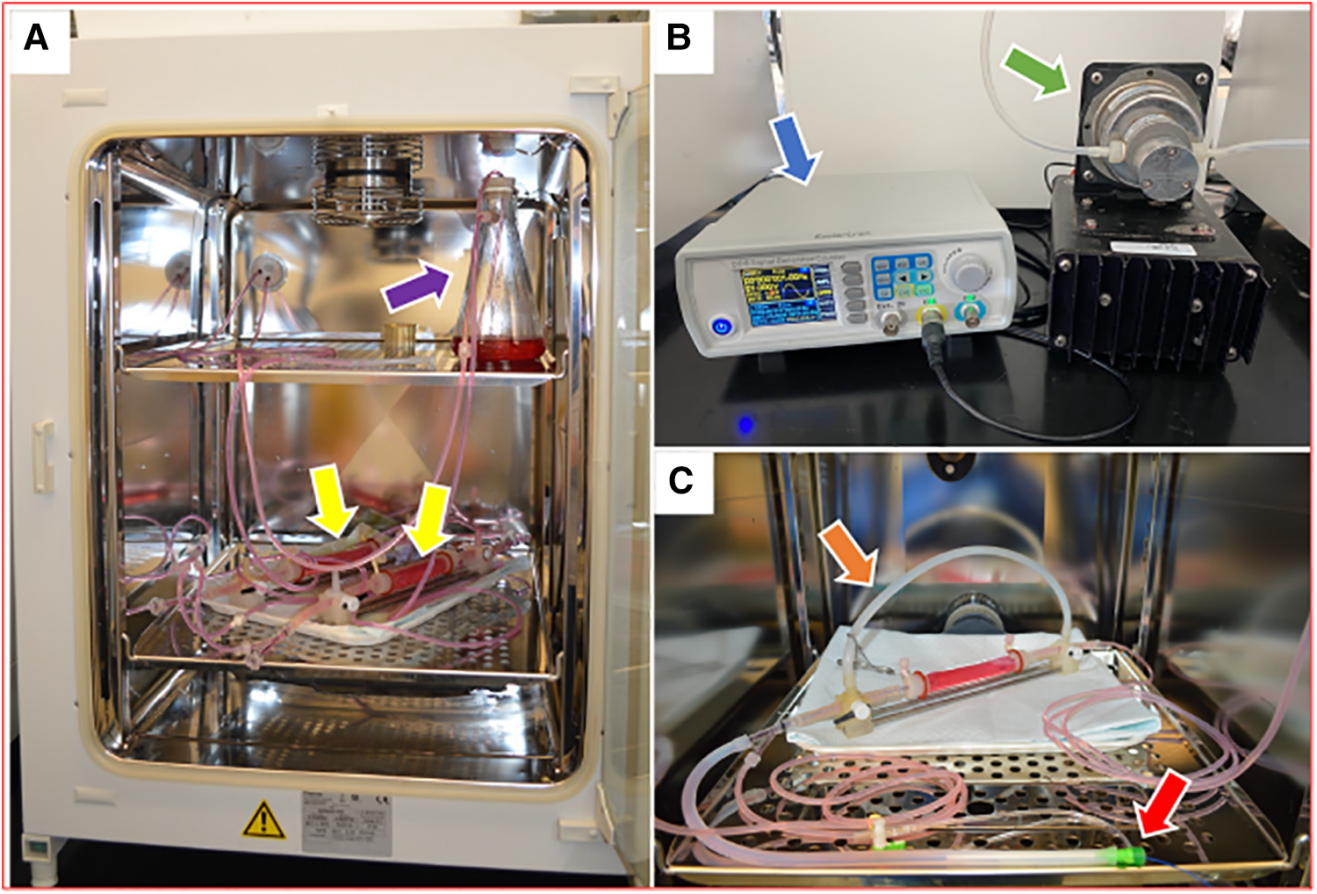
*Ex vivo* bioreactor system (**A**) bioreactor system is stored in an incubator and circulates culture medium through a closed loop from a medium reservoir (purple arrow) to artery housings (yellow arrows). (**B**) A signal generator (blue arrow) and a gear pump (green arrow) control the hemodynamic conditions in the bioreactor system. (**C**) A single artery housing with a bypass tube (orange arrow) and a balloon catheter that is inserted through a 6 Fr short sheath (red arrow).

The harvested porcine carotid arteries used in these studies were purchased from Animal Biotech Industries, Inc. Upon arrival, the arteries were rinsed in saline and excess fat, connective tissue, and fascia were dissected from each artery. The arteries were cut to lengths ranging from approximately 5–8 cm and stored in 15 ml centrifuge tubes at −20°C until needed. As described previously (8, 9), the *ex vivo* bioreactor system used in this study consisted of a flow reservoir, pump, vessel housing compartment, and distal flow constructor (Figure 1). The pressure was monitored by using a catheter pressure transducer (Millar Instruments, Houston, TX, USA). The flow was monitored by using an ultrasonic flow meter (Transonic Systems Inc., Ithaca, NY, USA). A signal generator (DDS Signal Generator Counter 15MHzm, Koolertron, Hong Kong) was used to generate pulsatile waveforms and to control and monitor the flow and pressure within the bioreactor system. The bioreactor flow medium consisted of DMEM containing low glucose [1,000 mg/L], 4.0 mmol/L L-glutamine, 110 mg/L sodium pyruvate, pyridoxine hydrochloride, 10% fetal bovine serum (Gibco), and 1% antibiotic-antimycotic (Gibco).

### Liquid drug delivery device comparisons

**Figure 2:**
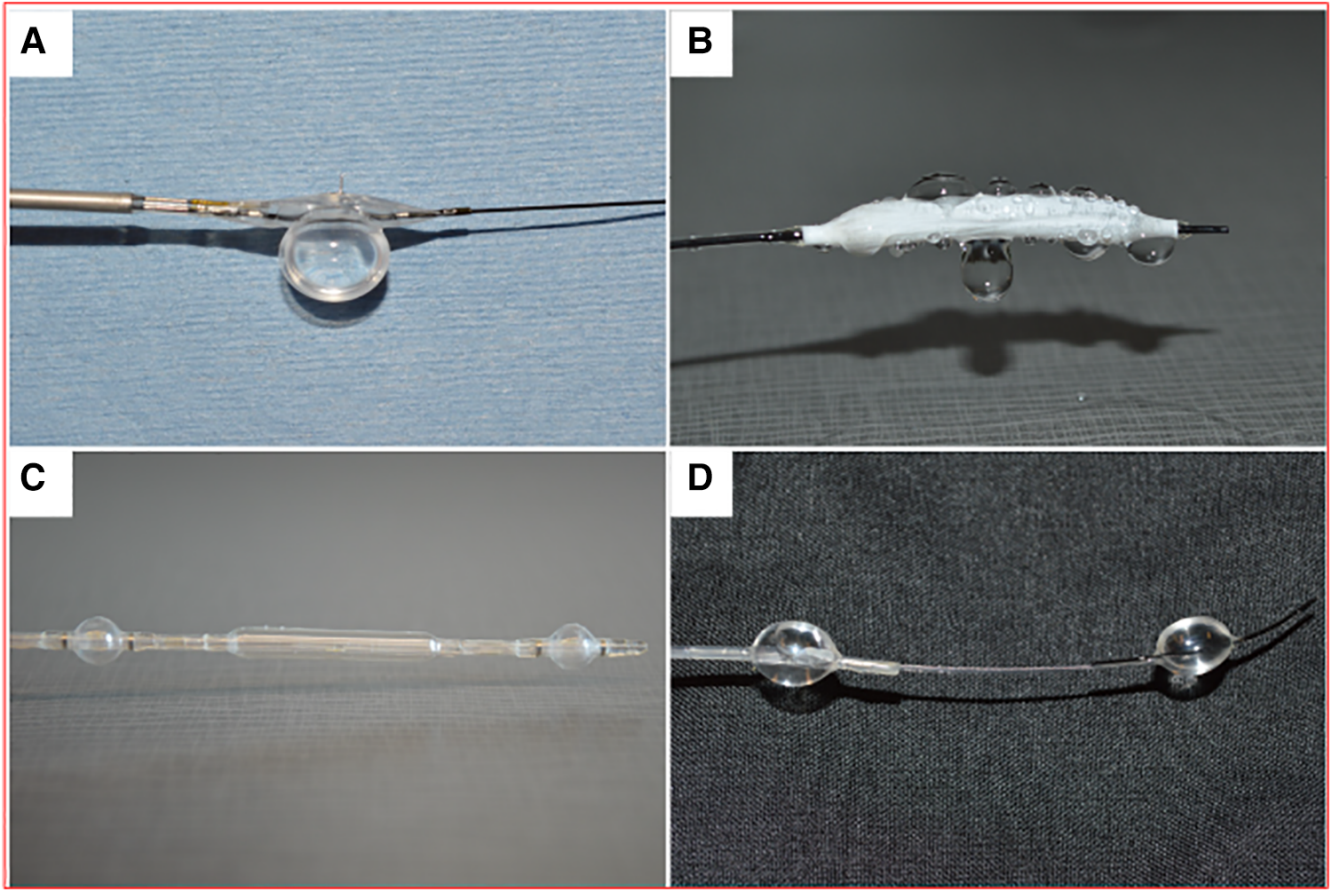
Liquid drug delivery devices. (**A**) Bullfrog catheter. (**B**) Clearway RX catheter. (**C**) Occlusion perfusion catheter (OPC). (**D**) targeted adjustable pharmaceutical administration system (TAPAS).

Four liquid drug delivery (LDD) devices were compared (Figure 2): the Bullfrog micro-infusion device (Mercator Medsystems, Emeryville, CA), the ClearWay RX catheter (Atrium Medical Corporation, Hudson, NH), the Occlusion perfusion catheter (OPC; Advanced Catheter Therapies, Chattanooga, TN, USA), and the targeted adjustable pharmaceutical administration system (TAPAS; Thermopeutix, San Diego, CA) (10). Performance of each device was compared by measuring the depth of penetration following 2 min of delivery of a fluorescent drug (Flutax-1, Tocris Bioscience, Bristol, UK) in the *ex vivo* bioreactor with a porcine carotid artery.

Flutax-1 (a fluorescent derivative of paclitaxel) was dissolved in DMSO and delivered at 0.1 mg/ml concentration in a 1:1 solution of saline (0.9% sodium chloride irrigation USP, Braun Medical Inc, Irvine, CA) and iohexol (Omnipaque, GE Healthcare, Marlborough, MA, USA). Delivery pressure of the Bullfrog catheter was achieved by low-pressure inflation (2 atm) to allow the microneedle to pierce into the vessel wall. Delivery through the ClearWay Catheter was performed at 4 atm. This low-pressure approach allowed the Flutax-1 to be delivered to the artery through the micropores. To deliver the drug through the OPC, the occlusion balloons were first deployed to their nominal size (4 atm). Using the pressure sensor within the treatment chamber, drug was delivered to achieve a treatment chamber pressure ranging from 0.2 to 0.4 atm. Delivery of the TAPAS catheter was achieved by inflations of the proximal and distal balloons using the recommended manufacturer inflation volume and delivering the liquid therapy through the infusion lumen at 2 atm. Delivery time for all devices was 120 s. The segments of the arteries treated with the drug were extracted after treatment for histological sectioning and fluorescent microscopy imaging to measure the depth of drug penetration.

### Histological sectioning and fluorescent microscopy imaging

Perfused carotid arteries were sectioned into 5-mm segments and frozen in optical cutting temperature (O.C.T.) compound (Sakura Finetek USA, Torrance, CA, USA). 12-micron cross-sections were cut using a cryostat. Sections were mounted on SuperFrost Plus Gold microscope slides (Fisher Scientific) using Prolong Diamond Anti-Fade Reagent (Invitrogen, Carlsbad, CA, USA) after washing with PBS. Sections were imaged with an Echo Revolve Fluorescence Microscope (Discover Echo, a BICO Company, San Diego, CA, USA) with a FITC filter to detect Flutax-1 fluorescence. Depth of drug penetration was measured from the internal elastic lamina to the maximum penetration depth and normalized to the thickness of the medial layer or wall thickness.

### Sirolimus and paclitaxel drug delivery

Solid sirolimus (R-5000 Rapamycin, LC Labs, Woburn, MA) was dissolved in 100% ethanol at a concentration of 6 mg/ml. Additionally, solid paclitaxel (P-9600 Paclitaxel, LC Labs, Woburn, MA) and liquid paclitaxel (Paclitaxel Injection USP, 6 mg/ml, Actavis Pharma, Parsippany, NJ, USA) were delivered to explanted pig arteries in the *ex vivo* model. The solid paclitaxel was also dissolved in 100% ethanol at a concentration of 6 mg/ml whereas the purchased liquid paclitaxel is pre-dissolved in polyoxyethylated castor oil. Each drug solution was diluted to 2.4 mg/ml with saline and iohexol using a 2:1:2 ratio by volume (2-part drug, 1-part saline, 2-part iohexol). The liquid sirolimus and paclitaxel were then delivered to the harvested porcine carotid artery using the OPC device, which was the only device selected for further evaluation based on the depth of penetration studies. Following 2 min of drug delivery at a 0.2–0.4 atm pressure, the device was removed, and the artery exposed to pulsatile flow conditions. At either 1 or 24 h post-treatment, the artery was removed for pharmacokinetic evaluation. Additionally, sirolimus coated balloons (SELUTION SLR, MedAlliance, Switzerland) with a drug density of 1 µg/mm^2^ were deployed at a balloon-to-artery ratio of 1.1:1 with an inflation time of 60 seconds and the treated arteries removed at 1-hour post-treatment for pharmacokinetic evaluation.

### Pharmacokinetic analysis

Perfused carotid artery segments were stored at −80°C and shipped on dry ice to the bioanalytical laboratory (iC42 Clinical Research and Development, Aurora, CO, USA). As previously described, quantification of arterial paclitaxel and sirolimus levels was performed using a validated high-performance liquid chromatography (HPLC)-electrospray ionization-tandem mass spectrometry system (LC-MS/MS) (9).

### Statistical analysis

All data are expressed as mean ± standard deviation (SD). Continuous variables in LDD comparisons were assessed with one-way analysis of variance (ANOVA) using GraphPad Prism 9 (GraphPad Software, La Jolla, CA, USA). Continuous variables in tissue drug concentrations were compared by using one-way and two-factor with replication ANOVA using GraphPad Prism 9. A value of *p* ≤ 0.05 was considered statistically significant. Tukey *post hoc* analyses were used to identify differences between groups if statistical differences were found with ANOVA.

## Results

### Fluorescent analysis

**Figure 3:**
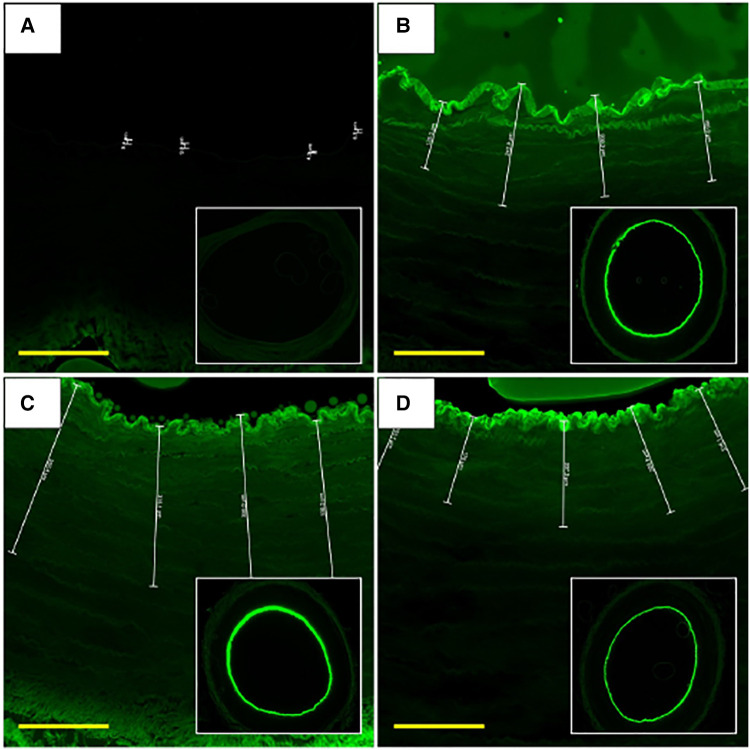
Fluorescent microscope images of Flutax-1 delivery with LDD devices at 0-hour. (**A**) Artery treated using Bullfrog catheter. (**B**) Artery treated using the Clearway RX catheter. (**C**) Artery treated using the OPC. (**D**) Artery treated using the TAPAS catheter. Scale bar = 180 µm. FITC filter. Images were taken using 2× and 10× objectives.

Each liquid delivery device was used to deliver a fluorescent liquid drug (Flutax-1) and the depth of drug penetration into the arterial tissue (*n* = 3 per device) was measured using fluorescence microscopy (Figure 3). Analysis of the images showed that drug delivery with the OPC had the deepest penetration of the drug at 233.65 ± 160.80 µm, with the TAPAS in second (127.22 ± 67.76 µm), followed by the ClearWay (117.55 ± 76.87 µm) and the Bullfrog (2.12 ± 3.78 µm). There was a trend towards differences in the average depth of penetration of Flutax-1 in the tissue samples between the four device groups (*p* = 0.098). Tukey *post hoc* analyses showed no significant differences between each of the four LDD systems, however there was a trend towards greater drug penetration with the OPC as compared to the Bullfrog (*p* = 0.069).

**Figure 4:**
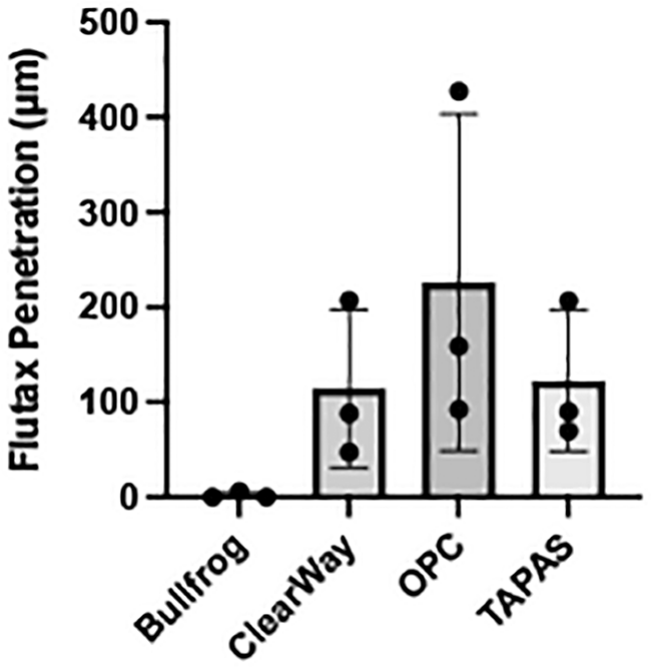
Average depth of penetration of Flutax-1 delivered with Bullfrog, ClearWay, OPC, and TAPAS liquid delivery devices.

The Bullfrog device punctured through the medial wall and delivered much of the Flutax-1 to the outer adventitial layer and into the bioreactor vessel housing, resulting in little retention of the drug in the medial wall of the artery. Among the ClearWay, OPC, and TAPAS devices, it was determined that Flutax-1 delivered via the OPC reached, on average, the deepest level of penetration within the vessel wall (Figure 4). Therefore, the OPC was selected as the LDD system for delivering sirolimus as a liquid drug.

### Pharmacokinetic analysis

**Figure 5:**
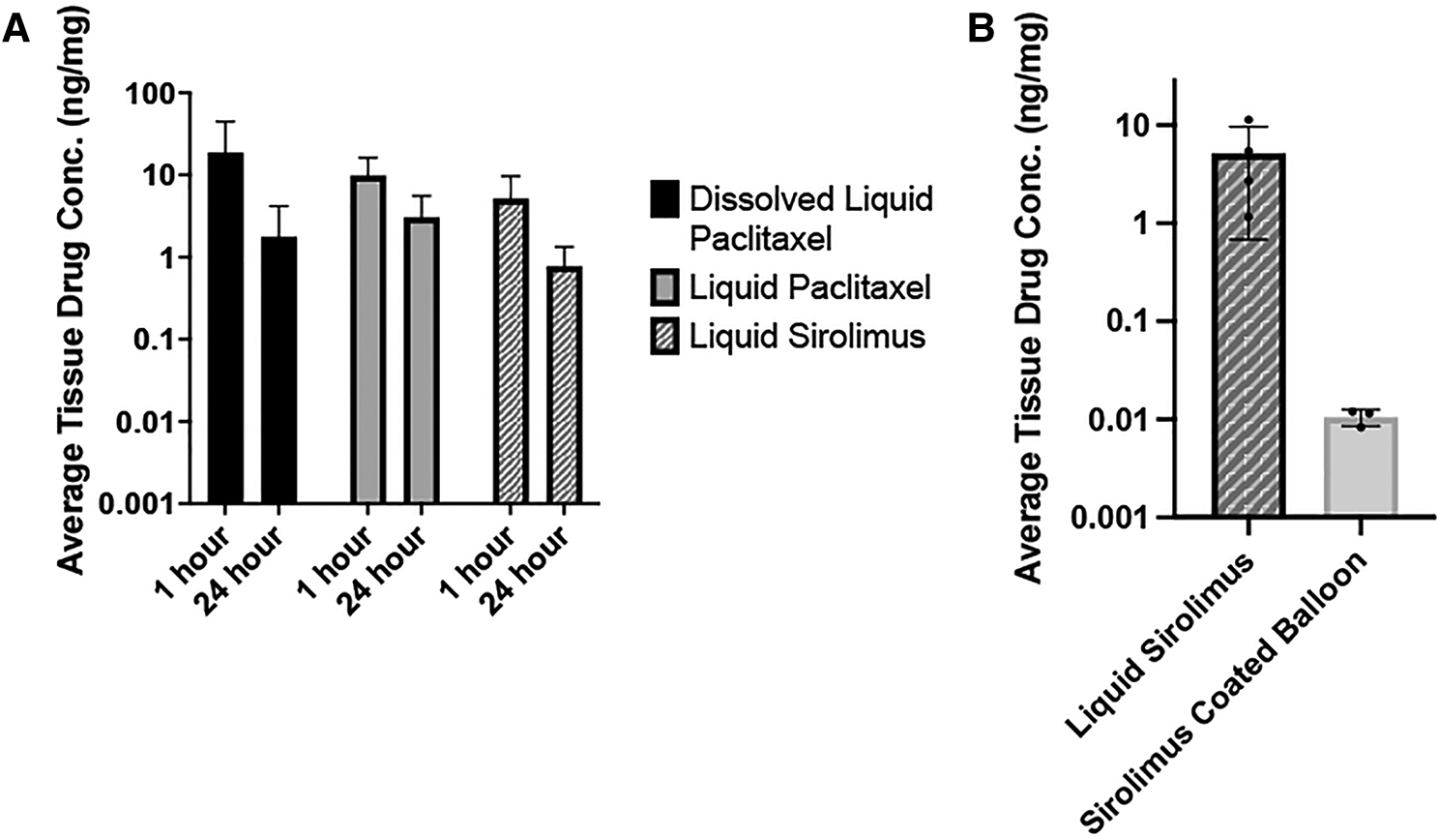
Pharmacokinetic analysis of sirolimus and paclitaxel treated arteries. (**A**) Average tissue drug concentration at 1- and 24-hour in arteries treated dissolved liquid paclitaxel, liquid paclitaxel, and liquid sirolimus. Drug solutions were delivered with the OPC. No significant differences were noted between any treatment groups or time points. (**B**) Average tissue drug concentration at 1-hour in arteries treated with liquid sirolimus versus sirolimus coated balloon.

Two time points—1- and 24-hour—were chosen to investigate retention of drug within vessel tissue, as the greatest drop-off in tissue drug retention occurs in the first 24 h after treatment (11). Figure 5 summarizes the drug concentrations (ng/mg) of the respective drugs in arterial tissue as detected by LC-MS/MS. The arteries treated with dissolved sirolimus retained 5.17 ± 4.48 ng/mg at 1-hour post-delivery and had a tissue drug concentration of 0.78 ± 0.55 ng/mg at 24-hour, showing an 84.86% decrease (*p* = 0.10). For comparison, arteries treated with dissolved paclitaxel retained 18.80 ± 25.61 ng/mg at 1-hour post-delivery and had 1.77 ± 2.41 ng/mg at 24-hour and had a 90.6% decrease (*p* = 0.23). Arteries treated with liquid paclitaxel retained 9.87 ± 6.28 ng/mg at 1-hour and 3.06 ± 2.50 ng/mg at 24-hour, which is a 69.02% decrease (*p* = 0.09). There was no statistical difference found between the three groups at either time point (*p* = 0.48 at 1-hour, *p* = 0.33 at 24-hour). Additional pharmacokinetic results demonstrated liquid sirolimus levels were higher than sirolimus coated balloons at 1-hour post-delivery (liquid sirolimus: 5.17 ± 4.48 ng/mg vs. sirolimus coated balloon: 0.0106 ± 0.002 ng/mg, *p* = 0.1096, Figure 5B).

## Discussion

In the 1990s, sirolimus was found to inhibit *in vitro* vascular smooth muscle cell proliferation and intimal thickening after balloon angioplasty and is the current drug of choice for coronary drug-eluting stents (12, 13). Even though limus-based drugs were adopted by industry as the main form of anti-proliferative drug for the treatment of coronary artery disease, paclitaxel has been the main drug to coat balloons and stents in the periphery. In 2018, however, a meta-analysis incited a reflection on the use of paclitaxel-coated balloons (PCB) in PAD, as a correlation was found between mortality rates and the PCB-treated patients at 1-, 2-, and 5-years (14). The FDA responded to the 2018 meta-analysis by conducting a review of their own and arrived at similar results, with a correlation between the use of PCBs and the mortality rates at 2- and 5-year follow-ups. Furthermore, in 2022, another meta-analysis was published discussing the elevated risk of amputation after PCB intervention, citing distal embolization of paclitaxel-excipient particles as a potential cause (15).

Paclitaxel, a taxane class drug, is cytotoxic and inhibits cell proliferation by interfering with microtubule dynamics during mitosis (16, 17). This drug is very lipophilic which allows for rapid delivery on PCBs. In contrast, sirolimus is cytostatic and blocks the mTOR kinase which is responsible for cell cycle progression from G1 to S phase. Additionally, while sirolimus is less lipophilic than paclitaxel, it has demonstrated anti-inflammatory effects, while paclitaxel has shown pro-inflammatory actions and toxicity at high doses which result in a lower therapeutic window than sirolimus (2, 3). Therefore, sirolimus has potential as an alternative therapeutic agent for PAD treatment.

The main objective of this study was to investigate the feasibility to locally deliver liquid sirolimus to targeted arterial lesions. We first evaluated the performance of four commercially available LDD devices in delivering a fluorescent analog of paclitaxel to the arterial wall. These included (1) the Bullfrog micro-infusion device, (2) ClearWay RX catheter, (3) Occlusion perfusion catheter (OPC), and (4) the targeted adjustable pharmaceutical administration system (TAPAS) (Table 1). Following these experiments, the OPC catheter was selected to deliver the liquid form of sirolimus into targeted arteries due to its performance in these initial studies. The results of the pharmacokinetic analysis demonstrated successful liquid delivery of sirolimus with the OPC and retention of the drug in porcine arterial tissue for up to 24 h.

**Table 1:** Summary of commercially available liquid drug delivery devices.

Device	Treatment chamber pressure	Device description	Guidewire compatibility	Size
Bullfrog micro-infusion device (Mercator Medsystems)	N/A	Microneedle for direct injection of drug upon inflation of device	0.014″	Diameter: 2–8 mm
ClearWay RX catheter (Atrium Medical Corporation)	N/A	Microporous PTFE low-pressure balloon for releasing liquid drug	0.014″ and 0.035″	Diameter: 1–4 mm; length 10–50 mm
Occlusion perfusion catheter (advanced catheter therapies)	0.2–0.4 atm	Proximal and distal occlusion balloons for isolating treatment zone; inflow and outflow port for infusing and removing drug; built-in fiber optic sensor for monitoring pressure in treatment zone	0.014″	Diameter: 3–7 mm; length: 80–150 mm
Targeted adjustable pharmaceutical administration system (Thermopeutix)	No sensor	Proximal and distal occlusion balloons for isolating treatment zone; adjustable treatment zone	0.014″	Diameter: 4.5–8.0 mm; length: 10–40 mm

N/A, not applicable; No sensor, no pressure sensor to monitor treatment chamber pressure; PTFE, polytetrafluoroethylene.

There are currently four sirolimus-coated balloons (SCB) that have received FDA breakthrough device designation for treatment of PAD: SELUTION SLR (Med Alliance), MagicTouch PTA (Concept Medical), SUNDANCE (Surmodics), and Virtue SAB (Orchestra BioMed) (Table 2). SELUTION delivers sirolimus at 1 µg/mm^2^ using poly(lactic-co-glycolic acid)-encapsulated sirolimus microspheres that are adhered to an angioplasty balloon with their Cell Adherent Technology (18). MagicTouch delivers a sirolimus dose of 1.27 µg/mm^2^ through their Nanolute technology, which features sirolimus nanoparticles enveloped in a phospholipid layer and containing a calcium phosphorus-core (19). The core allows for pH-sensitive release of the encapsulated drug while the hydrophobic exterior improves tissue uptake of sirolimus. The Surmodics Sundance sirolimus DCB utilizes a crystalline drug release platform to deliver sirolimus. While the drug dosage has yet to be disclosed, preclinical pharmacokinetic analysis of the Sundance DCB revealed higher tissue sirolimus concentrations than SELUTION SLR and MagicTouch PTA and remained above the therapeutic threshold of 1 ng/mg at 90 days. Lastly, the Orchestra BioMed Virtue is a sirolimus eluting balloon that uses AngioInfusion technology to deliver an extended focal release formulation of sirolimus (SirolimusEFR) through micropores in the angioplasty balloon (20). Sirolimus submicron particles are first reconstituted in an aqueous solution to a specified dosage and dispersed with the inflation of the balloon.

**Table 2:** Summary of sirolimus-coated balloons that have received FDA breakthrough device designation for PAD use.

Devices	Dose	Drug carrier	Guidewire compatibility	Size
SELUTION SLR (Med Alliance)	1 µg/mm^2^	PLGA-encapsulated sirolimus microspheres (Cell Adherent Technology)	0.014″, 0.018″	Diameter: 1.5–5 mm; length: 10–40 mm
MagicTouch PTA (Concept Medical)	1.27 µg/mm^2^	Phospholipid-based excipient (Nanolute)	0.014″, 0.018″, 0.035″	Diameter: 1.5–12 mm; length: 10–200 mm
SUNDANCE (Surmodics)	Not reported	Microcrystalline drug release platform	0.014″	Diameter: 2–4 mm; length: 20–220 mm
Virtue SAB (Orchestra BioMed)	3 mg	Sirolimus submicron particles (SirolimusEFR) delivered in aqueous solution	0.014″	Not reported

PLGA, poly(lactic-co-glycolic acid).

It was noteworthy that the four tested devices led to different penetration results. This is likely due to the different mechanisms by which these devices work. The Bullfrog is designed for injection of the therapeutic drug into the adventitial layer of vessels. This device has a microneedle that extends with low-pressure (2 atm) inflation. The ClearWay infuses drug into the tissue from micropores in the low-pressure balloon, similar to the Virtue balloon. The device utilizes occlusion, containment, and infusion (OPI) therapeutics to localize and contain the treatment. The OPC is a multi-luminal device with proximal and distal occlusion balloons, which restrict blood flow into the treatment region and allow for pressurized delivery of liquid drug, and in- and out-flow ports that allow blood and excess drug to be flushed from the treatment region. This device also has a built-in fiber optic pressure sensor to monitor the pressure in the treatment region. Finally, the TAPAS, another multi-luminal device, has proximal and distal occlusion balloons that can be adjusted to better fit the lesion length and a pressure lumen that allows the pressure in the treatment zone to be monitored. To assess these four devices, our initial study utilized a fluorescent paclitaxel (Flutax-1) due to the ability to readily detect the distribution of the drug and due to a lack of a fluorescent sirolimus analog. Our results demonstrated that, of these four devices, the OPC was able to deliver the fluorescent drug deepest into the vascular layers. Therefore, it was selected as the investigation device for delivering liquid sirolimus.

The liquid delivery approach has many potential advantages over balloon-based drug therapy and drug eluting stents. LDD devices, such as the OPC, can administer liquid drug deep into target tissue, with the depth of penetration correlating with the delivery pressure (21, 22). This allows for drug to be delivered directly to the medial layer of arteries as opposed to diffusing from drug-excipient particles adhered to the endothelium. Another benefit of this pressurized delivery of liquid drugs is the decreased risk of particulate distal embolization. This is a common issue with crystalline balloon coatings and has been hypothesized to be a factor in the high correlation of death and amputation associated with PCB use in PAD (15). Additionally, without the use of a solid, crystalline coating, there is reduced loss of drug during the tracking of the device to the lesion. Another advantage of LDD is economical, as an LDD device can be used more than once to treat very long or multiple lesions. Lastly, LDD devices can be used to test alternative therapeutic agents. The OPC has been used in preclinical trials to investigate the treatment efficacy of a vascular smooth muscle cell-targeting molecule, revealing successful reduction of neointimal growth in benchtop and porcine models (22, 23). However, there are disadvantage of LDD. While DCBs come preloaded with the drug, LDDs require the additional step of preparing the liquid therapy. Treatment at branching or bifurcations are more challenging with liquid delivery, given the solution can potentially leave the targeted treatment zone. To overcome this challenge, the end balloon of the catheters can be placed strategically at the bifurcation to “cover” the branch and minimize liquid drug loss. Additionally, with devices such as the OPC and the TAPAS, the delivery pressure must be maintained in the treatment zone to allow the drug to infuse into the tissue. The OPC has a built-in sensor for such purposes, while other delivery devices are not equipped.

In comparison to paclitaxel, the liquid sirolimus showed similar levels at 1-hour to both dissolved paclitaxel and liquid paclitaxel (liquid sirolimus: 5.17 ± 4.48 ng/mg vs. dissolved paclitaxel: 18.80 ± 25.61 ng/mg vs. liquid paclitaxel: 9.87 ± 6.28 ng/mg, *p* = 0.47) and above the therapeutic threshold of 1 ng/mg. At 24-hour, we observed a similar trend, with no differences between the groups (liquid sirolimus: 0.78 ± 0.55 ng/mg vs. dissolved paclitaxel: 1.77 ± 2.40 ng/mg vs. liquid paclitaxel: 3.06 ± 2.50 ng/mg, *p* = 0.47). And although the differences between the three groups were not statistically significant, lower retention of drug was observed in the dissolved sirolimus group compared to the dissolved and liquid paclitaxel groups. This was somewhat expected, as sirolimus has poorer lipophilicity and bioavailability than paclitaxel (24, 25). Based on these results, future studies will incorporate appropriate excipients to improve tissue retention of sirolimus and decrease the drug drop-off over time.

To allow a direct comparison of the liquid delivery of sirolimus with the OPC device, we deployed a commercially available sirolimus coated balloon in the *ex vivo* bioreactor system and analyzed for pharmacokinetic data. The drug tissue concentration of the DCB-treated arteries was found to be lower than the liquid sirolimus-treater arteries at 1-hour post treatment (Sirolimus coated balloon: 0.0106 ± 0.002 ng/mg vs. liquid sirolimus: 5.17 ± 4.48 ng/mg, *p* = 0.1096). This result further confirms the promising delivery approach in targeting arteries with liquid sirolimus.

It is worth noting some limitations in this study. First of all, we utilized healthy porcine arteries that lack the branching and bifurcations of native arteries. Diseased arteries are more complex having intimal thickening, fibrosis, and calcification, all of which may affect the diffusion of the drug, and arteries free of bifurcations may not accurately represent target vessels in clinical settings. Additionally, our system lacks blood, which may impact drug delivery and retention, although these differences are more severe for longer duration *ex vivo* studies.

## Conclusion

In this study, we demonstrated for the first time the feasibility to deliver liquid sirolimus into the arterial wall. Four commercially available liquid delivery devices were evaluated for their ability to penetrate liquid drug into the vessel. Fluorescence microscopy demonstrated that drug delivery with the OPC had greater drug penetration into the medial wall as compared to other devices. Liquid sirolimus delivery versus liquid paclitaxel was then evaluated by the selected OPC device. Liquid sirolimus showed similar drug levels within the target tissue as compared to liquid paclitaxel, but greater than a commercially available sirolimus coated balloon. With growing concerns regarding the safety of paclitaxel-coated balloons in peripheral artery disease use, liquid delivery of sirolimus is potentially an innovative approach to treat occluding peripheral lesions. Further studies are warranted to show excipients can improve sirolimus retention and that the therapy can inhibit neointimal growth and re-occlusion *in vivo*.

## Data Availability

The raw data supporting the conclusions of this article will be made available by the authors, without undue reservation.
